# Pathogenic Potential of *Pseudoxanthomonas kaohsiungensis* Strain IMB-1 Based on Whole-Genome Sequencing

**DOI:** 10.3390/biology15131010

**Published:** 2026-06-25

**Authors:** Natalia Belkova, Nadezhda Smurova, Raisa Zugeeva, Elizaveta Klimenko, Ekaterina Grigorova, Marina Dorzhieva, Uliana Nemchenko

**Affiliations:** Federal State Budgetary Scientific Institution ‘Scientific Centre for Family Health and Human Reproduction Problems’, Epidemiology and Microbiology Institute, 3, K. Marks Str., 664003 Irkutsk, Russia; nadinasmurova@mail.ru (N.S.); raya.zugeeva@mail.ru (R.Z.); klimenko.elizabet@gmail.com (E.K.); buxarowa.ekaterina@yandex.ru (E.G.); zdravst8y1te@gmail.com (M.D.); umnemch@mail.ru (U.N.)

**Keywords:** *Pseudoxanthomonas kaohsiungensis*, resistance phenotype, ribosomal taxonomy, whole genome sequencing, dDDH, phylogenetic analysis, antibiotic resistance, virulence, efflux pump

## Abstract

Mass spectrometry and high-throughput sequencing have been introduced into clinical bacteriology. We characterized strain IMB-1, previously isolated from the cerebrospinal fluid of a child, as *Pseudoxanthomonas kaohsiungensis* and analyzed its biological properties, resistance phenotype, and complete genome. The IMB-1 strain displayed amylolytic, weak lipolytic activities, and it exhibited a phenotypic resistance profile only for aminoglycosides. The dDDH calculation based on the complete genome sequence showed that strain IMB-1 was closely grouped with the type strain *P. kaohsiungensis* DSM 17583, and the dDDH (d4) value was 70.1%. A comparative pan-genome analysis was performed for four *P. kaohsiungensis* genomes, revealing a substantial shared core genome. The IMB-1 genome contained 508 unique gene clusters, representing the largest strain-specific gene set among the analyzed genomes, suggesting genomic plasticity and adaptation to the host-associated environment. Genome annotation revealed genes responsible for antibiotic, disinfecting agent, and antiseptic resistance. Gene clusters exhibiting the potential to form biofilms, adhere to the epithelial surface, and exhibit resistance to stress factors were identified. Our study demonstrates that strain IMB-1 is a potential opportunistic pathogen with significant pathogenic potential. The application of high-resolution whole-genome sequencing data in public health for pathogen identification and monitoring can improve the accuracy of infection source determination, reduce the scale and burden of outbreaks, and identify and quantify antimicrobial resistance in pathogens.

## Simple Summary

The IMB-1 strain was isolated from the cerebrospinal fluid of a child. We identified it as *Pseudoxanthomonas kaohsiungensis* and analyzed its biological properties, resistance phenotype, and complete genome. The *P. kaohsiungensis* strain IMB-1 displayed amylolytic and weak lipolytic activities, and it exhibited a phenotypic resistance profile only for aminoglycosides. A comparative pan-genome analysis revealed a large, conserved core and a diverse accessory genome, with IMB-1 exhibiting the highest proportion of unique genes. Genomic data analysis indicated virulence potential, which was confirmed by the presence of the type I secretion system (TISS), type IV pili, genes responsible for flagellar and isocitrate lyase (ICL) biosynthesis, and resistance potential to antibiotics, disinfectants, and antiseptics.

## 1. Introduction

Modern technologies, especially mass spectrometry and high-throughput sequencing, have been introduced into clinical bacteriology. They have greatly expanded our knowledge of opportunistic bacteria. These tools allowed the identification of rare and previously unknown species that can cause human diseases [1,2,3,4,5,6]. The description of new bacterial species that were not previously classified as human pathogens is a priority in medical microbiology. Some of these bacterial species have two-faced biological characteristics: clinical cases involving them are registered, and some strains of these species are actively used in biotechnology [7,8]. However, cases are increasingly being described where natural species, primarily found in soil and water, realize their pathogenic potential in altered and stressful environmental conditions when in contact with animals or humans. Such environmental and commensal species with clinical significance have been described among Gram-positive and Gram-negative bacteria [6,9,10]. In immunocompromised patients, they most often cause infections, and patient comorbidities and a delay in pathogen identification are among the factors likely contributing to the development of infection [1,11]. Diagnostic difficulties highlight the importance of careful interpretation of microbiological test results and awareness of rare microorganisms in cases where standard therapy is ineffective.

The genus *Pseudoxanthomonas* was first characterized in 2000 [12]. Most species of this genus were first isolated and described from water, plants, and contaminated soils [13,14,15,16]. Among the yellow-pigmented isolates, the type strain of the genus *Pseudoxanthomonas, Pseudoxanthomonas broegbernensis* B1616/1^T^, was isolated from experimental biofilters used for the waste gas treatment of an animal-rendering plant. The isolate was characterized by its ability to degrade nitrite without producing nitrogen gas; it did not reduce nitrate, and its final product was nitrous oxide. The strain was resistant to erythromycin, streptomycin, nalidixic acid, kanamycin, ampicillin, penicillin G, gentamicin, fucidin, tetracycline, and novobiocin [12]. *Pseudoxanthomonas taiwanensis* CB-226^T^ was isolated from hot springs in the Chi-ban area, Taitung, Taiwan, and characterized by an optimum growth temperature of 50–60 °C, unusual denitrification reaction, reducing nitrite, but not nitrate, with the production of N_2_O only, and absence of flagella [13].

However, isolates of only some species of this genus have been derived from clinical material of patients and have been reported to be of clinical significance [1,14,17]. *Pseudoxanthomonas mexicana* was previously isolated from the urine of a 10-year-old boy in Germany with multiple catheterizations and occasional urinary tract infections [14]. A strain of the *Pseudoxanthomonas kaohsiungensis* species was isolated from the blood of a worker in an oil refinery in Taiwan [1]. Twelve isolates of the *Pseudoxanthomonas* genus were isolated from 10 patients at different hospitals in Canada over 7 years and were identified as *Pseudoxanthomonas winnipegensis* [17]. In addition, strains of the *Pseudoxanthomonas* genus have been isolated from swabs in a ward for patients with cystic fibrosis, although the authors believe that these strains have no particular clinical significance for these patients [18]. Bacteria belonging to this genus were identified by 16S rRNA gene amplicon metagenomics and had been associated with various diseases, serving as microbiota-associated markers of human health [19,20,21]. Thus, in a study by Riquelme E. and co-authors, *Pseudoxanthomonas, Streptomyces, Saccharopolyspora*, and *Bacillus clausii* were identified as an intratumoral microbiome signature highly predictive of long-term survivorship in patients with pancreatic adenocarcinoma [19]. Xu H. and co-authors showed that the genus *Pseudoxanthomonas* in salivary microbiota might be related to tonsillar hypertrophy [20]. Zhu Y. and co-authors studied the pancreatic microbiota and found that *Pseudoxanthomonas* was decreased in the tissues of patients with pancreatic cancer [21].

Despite the fact that, among the species of the genus *Pseudoxanthomonas*, only strains of three species were derived from human clinical materials and characterized in detail [1,14,17], representatives of this genus are detected by molecular methods as microbiota-associated markers of human diseases [19,20,21]. These results highlight the urgent need to use genomic data to identify microorganisms and uncover the genetic potential of emerging pathogens for drug resistance. This study aimed to characterize the biochemical properties and resistance phenotype of the IMB-1 strain, identify its protein spectrum and ribosomal taxonomy, perform phylogenomic analysis, and characterize its pathogenic potential using genomic data.

## 2. Materials and Methods

### 2.1. Object of the Study

The IMB-1 strain was used in this study. It was deposed to the working collection of the Microbiome and Microecology Laboratory at the Scientific Center for Family Health and Human Reproduction Problems (SC FHHRP, Irkutsk, Russia) from the bacteriology laboratory of the Ivano-Matreninskaya City Children’s Clinical Hospital in Irkutsk. The strain was previously isolated from the child’s cerebrospinal fluid and identified as oxidase–catalase positive, non-fermenting Gram-negative bacteria (NFGNB) using the NEFERMtest 24 test system manufactured by Erba Lachema (Brno, Czech Republic).

### 2.2. Determination of the Biochemical Properties

The activities of extracellular amylase, lipase, and protease enzymes were determined according to a previously described method [22].

A starch-containing medium was prepared to detect the amylolytic activity. The medium was sterilized at 1.0 atm and poured into sterile Petri dishes. Bacterial cell culture was streaked onto the solidified medium; *Staphylococcus aureus* strain ATCC 25923 served as a control. The culture was incubated for 48 h. To detect starch hydrolysis, 3 mL of Lugol’s iodine solution (Research Center of Pharmacology, St. Petersburg, Russia) was poured onto the surface of the medium. The starch-containing medium turned blue, whereas the hydrolysis zone remained colorless. The starch hydrolysis zone was measured in millimeters from the edge of the streak to the edge of the light zone. A larger light zone diameter indicates higher amylolytic activity.

To determine proteolytic activity, meat-peptone gelatin (MPG) was prepared and dispensed into 8–10 mL test tubes. The tubes were sterilized at 0.5 atm for 15 min. The cultures were inoculated deep into the medium column by pricking and left at room temperature for 2 days. The gelatin liquefaction was visually noted.

Lipolytic activity was determined by plating on brain heart agar (BHA, HiMedia, Thane, India) supplemented with 0.1% Tween-80 and 10 mM CaCl_2_ (final concentrations). After incubation at 37 °C for 48 h, the plates were incubated at 4 °C for 24 h. Exogenous lipase activity was assessed by the formation of a “halo” of insoluble calcium salts of free fatty acids around the colonies. A *Corynebacterium kefirresidentii* strain with confirmed lipase activity was used for comparison [23].

To determine the ability to form biofilms, a 24-h culture of *P. kaohsiungensis* with a density of 0.5 according to the McFarland turbidity standard, which approximately corresponds to a load of 1–2 × 10^8^ CFU/mL, and a dilution of 100-fold to 1 × 10^6^ CFU/mL was used. Biofilms were stained according to the method of O’Toole G.A. [24]. The intensity of biofilm formation was assessed by the level of ethanol extraction of the dye at a wavelength of 492 nm in the optical density units (OD492). The measurement results were interpreted by comparing the OD492 values of the samples with those of the negative control (pure solvent without added dye). The presence of biofilm was recorded when the OD492 value of the sample was greater than or equal to that of the control. All experiments were carried out in three technical replicates.

The minimum inhibitory concentration (MIC) was determined using the MIC NEFERM and MIC G-II tests (SENSILATEST, Erba Lachema s r.o., Brno, Czech Republic) to assess the antibiotic susceptibility of NFGNB and Enterobacteriaceae. This was based on the analysis of bacterial growth at breakpoint concentrations of the antibiotic, in accordance with the EUCAST standards. The tests contained the following antibiotics: ampicillin/sulbactam (ams), piperacillin (pip), piperacillin/tazobactam (pit), ceftazidime (caz), aztreonam (azt), meropenem (mer), gentamicin (gen), amikacin (amk), colistin (col), ciprofloxacin (cip), tigecycline (tgc), trimethoprim/sulfamethoxazole (t/s), cefotaxime (ctx), cefoperazone (cpz), cefoperazone/sulbactam (cps), cefepime (cep), ertapenem (ert), netilmicin (net), and tobramycin (tob). A 100 μL bacterial suspension with a turbidity of 0.5 according to McFarland’s was inoculated into microwells of strips and incubated at 37 °C. The results were considered according to the manufacturer’s instructions after 20 and 72 h.

### 2.3. MALDI-TOF Identification

Preliminary profiling identified the IMB-1 strain as *Stenotrophomonas maltophilia*. To confirm the identification and analyze the homology of protein spectra, *S. maltophilia* strains 43 and 75 from the collection of the SC FHHRP laboratory (Irkutsk, Russia) were used for future study. All strains were grown on meat-peptone agar for protein profiling. Cell biomass of the analyzed strains from a single colony was suspended in 150 μL of deionized water. 450 μL of 96% ethanol was added to the resulting suspension, and the mixture was centrifuged at 13,400 rpm for 2 min. The supernatant was removed, and 10 μL of 70% formic acid was added to the pellet. The mixture was mixed until completely dissolved. An equal volume of acetonitrile was added to the resulting solution, mixed gently, and centrifuged at 13,400 rpm for 2 min. The supernatant was transferred to a new tube for analysis.

To prepare the target for protein profiling, a commercial “MALDI-TOF Proba” kit (Litech, Moscow, Russia) was used. One microliter of supernatant was added to the target well, air-dried, and then 1 microliter of matrix (α-cyano-4-hydroxycinnamic acid) was added. Analysis was performed on a Smart MS 5020 instrument (Zhuhai DL Biotech, Zhuhai, China) in the mass range of 3000–20,000 Da with primary identification according to the instrument database (v. 20250409). The obtained spectra were processed using the specialized software Microbe Analysis v.1.1.1.1.

### 2.4. Molecular Analysis

Genomic DNA was isolated from a 24-h culture of the IMB-1 strain using a commercial DNA-sorb-B kit (AmpliSens^®^, Moscow, Russia). Several colonies were streaked from the surface of the agar medium, and the cell biomass was resuspended in 100 µL of sterile saline and lysed at 65 °C for 5 min. DNA was precipitated on the sorbent, followed by three washes to remove unbound components. The purified DNA was eluted in TE buffer.

The nearly complete 16S rRNA gene sequence was amplified by PCR using conservative bacterial primers. The PCR mixture was prepared using the Encyclo Polymerase kit (Eurogen, Moscow, Russia), which includes a 50× mix of thermostable DNA polymerases with a hot start and 10× Encyclo buffer. Deoxynucleoside triphosphates were added to the reaction to a final concentration of 0.2 mM each, primers to 1 pM, and template DNA to 0.5 ng/μL. Amplification was performed on a DTprime cycler (DNA-Technology, Moscow, Russia) in the following mode: initial denaturation at 95 °C for 5 min; then 25 cycles (94 °C for 30 s, 60 °C for 30 s, 72 °C for 30–60 s); final elongation at 72 °C for 5 min. The PCR products were purified after gel electrophoresis in 1% agarose gel with the addition of ethidium bromide (10 mg/mL) and subsequent visualization on a transilluminator. We used the Brilliant Dye Cycle Sequencing Kit v. 3.1 (NimaGen, Nijmegen, The Netherlands) for sequencing. The reaction included an initial step at 96 °C (45 s), followed by 25 cycles (96 °C for 10 s, 50 °C for 5 s, and 60 °C for 4 min). Amplicons were purified by ethanol precipitation and analyzed by capillary electrophoresis on a Nanofor-05 genetic analyzer (Synthol, Moscow, Russia).

### 2.5. Ribosomal Taxonomy

Nucleotide sequence editing and assembly of the complete 16S rRNA gene were performed manually using the Bioedit v.7.7 software environment [25]. The species affiliation of the strain was determined by comparing the obtained nucleotide sequence with the NCBI (National Center for Biotechnology Information) “16S ribosomal RNA sequences (Bacteria and Archaea)” database using the BLAST+ v2.16.0 [26,27].

The 16S rRNA gene sequences of the type strains of 27 representatives of the genus *Pseudoxanthomonas* were selected from the List of Prokaryotic Names with Standing in Nomenclature (LPSN) database for phylogenetic analysis [28,29]. Additionally, sequences of the species *P. beigongshangi*, *P. broegbernensis*, *P. daejeonensis*, *P. humi*, *P. icgebensi*, *P. indica*, *P. jiangsuensis*, *P. kaohsiungensis*, *P. koreensis*, *P. mexicana*, *P. sacheonensis*, *P. taiwanensis*, *Pseudoxanthomonas* sp., as well as sequences of uncultured clones of *Pseudoxanthomonas* sp. longer than 1400 bp, were included from the NCBI database [30].

Multiple sequence alignment was performed using the ClustalW algorithm in MEGA v.12 [31]. Phylogenetic trees were constructed using the same program and the neighbor-joining (NJ) method [32]. The reliability of the topology was assessed using 1000 replicates of bootstrap analysis. The 16S rRNA gene sequence of *Bacillus subtilis* IAM 12118 (AB042061) was used as an outgroup for tree rooting.

### 2.6. Whole-Genome Sequencing

For whole-genome sequencing, DNA with a concentration of >4 ng/μL was used. The MGI G400 platform (MGI, Shenzhen, China) was used for sequencing using the MGIEasy Fast FS Library Prep Set (FCL PE150) (MGI, Shenzhen, China). For long-read sequencing, DNA with a concentration of 70 ng/μL was used. Sequencing was performed on the PromethION platform (Oxford Nanopore Technologies, Oxford, UK) at the Core Facility Center «Genomics, Proteomics and Metabolomics» at Lopukhin Federal Research and Clinical Center of Physical-Chemical Medicine of Federal Medical Biological Agency (Moscow, Russia).

### 2.7. Bioinformatic Analysis

Trim Galore v.0.6.10 was used to remove adapters and low-quality sequences from Illumina paired-end reads v.0.6.10 [33]. Nanopore reads were filtered by quality and length using Filtlong v.0.3.1 [34], and sequencing statistics were assessed using NanoStat v.1.6.0 [35]. De novo genome assembly was performed from Oxford Nanopore long reads using Flye v.2.9.6 [36]. The resulting assembly was polished by aligning Nanopore reads with minimap2 v.2.31 [37] followed by consensus correction using Racon v.1.5.0 and Medaka v.2.2.1 [38]. Illumina short reads were subsequently used for additional polishing with Racon v.1.5.0 [39]. The final assembly resulted in a single circular chromosome. Assembly quality and genome statistics were evaluated using QUAST v.5.3.0 [40]. Technical contamination was identified and removed using NCBI FCS-GX v.0.5.5 [41].

The functional annotation was performed using Prokka v.1.14.6 [42]. Bacterial genes were annotated using eggNOG Mapper v.5.0.2 [43], and the Kyoto Encyclopedia of Genes and Genomes (KEGG) was annotated using the BlastKOALA v.3.1 server [44]. The completeness of the KEGG pathway was calculated using the KEGG-pathways-completeness tool v.1.3.0 [45].

Prophage sequence identification and annotation were performed using the PHASTER web server (accessed on 18 May 2026) in the complete genome [46]. Genome alignment factors (AF) and average nucleotide identity (ANI) values were calculated using the fastANI method [47]. Roary v.3.13.0 was used to compare the gene landscape [48].

Genes associated with antibiotic resistance were searched using standard settings in the CARD v.4.0.1 [49] and ResFinder v.4.7.2 [50] databases.

Virulence genes were detected using the reference database for bacterial virulence factors (VFDB) [51] with a homology threshold of 80%. We used the full VFDB dataset [52], which contains all genes associated with known and putative virulence factors. Virulence factors with a VFDB sequence alignment length of >90 nucleotides were selected for further analysis.

### 2.8. Phylogenetic Analysis

The genome sequence data of strain IMB-1 were uploaded to the Type (Strain) Genome Server (TYGS), a free bioinformatics platform for whole-genome-based taxonomic analysis (https://tygs.dsmz.de, 11 June 2026) [53]. The analysis also used recently introduced methodological updates and features [29,54]. Information on nomenclature, synonymy, and associated taxonomic literature was provided by TYGS’s sister database, the LPSN [28,29,55]. The results were provided by the TYGS on 11 June 2026. The TYGS analysis was subdivided into the following steps:

(1) Determination of closely related type strains

The closest type strain genomes were obtained in two complementary ways: First, all user genomes were compared against all type strain genomes available in the TYGS database using the MASH algorithm, a fast approximation of intergenomic relatedness [56], and the ten type strains with the smallest MASH distances were chosen per user genome. Second, an additional set of ten closely related type strains was determined using 16S rDNA gene sequences. These were extracted from the user genomes using RNAmmer [57], and each sequence was subsequently treated using BLAST+ v2.16.0 [58] against the 16S rDNA gene sequence of each of the currently 24,481 type strains available in the TYGS database. This was used as a proxy to identify the 50 best-matching type strains (by bitscore) for each user genome and to subsequently calculate precise distances using the Genome BLAST Distance Phylogeny (GBDP) algorithm with the ‘coverage’ distance formula d5 [59]. Finally, these distances were used to determine the 10 closest type-strain genomes for each user genome.

(2) Pairwise comparison of the genome sequences

For phylogenomic inference, all pairwise comparisons among the set of genomes were conducted using GBDP and accurate intergenomic distances inferred under the algorithm ‘trimming’ and distance formula d5 [59]. 100 distance replicates were calculated. Digital DDH values and confidence intervals were calculated using the recommended settings of the GGDC 4.0 [55,59].

(3) Phylogenetic inference

The resulting intergenomic distances were used to infer a balanced minimum-evolution tree with branch support via FASTME 2.1.6.1, including SPR post-processing [60]. Branch support was inferred from 100 pseudobootstrap replicates each. The trees were rooted at the midpoint [61] and visualized with PhyD3 [62].

(4) Type-based species and subspecies clustering

The type-based species clustering using a 70% dDDH radius around each of the 31 type strains was performed as previously described [54]. Subspecies clustering was performed using a 79% dDDH threshold as previously described [63].

### 2.9. Validation of the WGS-Based Taxonomy

The WGS-based taxonomy was validated by computing ANIb values for all pairwise genome comparisons using JSpeciesWS (accessed on 14 April 2026) [64]. Only genomes satisfying two criteria were included in the validation: (1) they are designated as reference strains in the NCBI Genome database, and (2) they belong to species of the genus *Pseudoxanthomonas* that have been validly published. Because ANIb values were asymmetric in pairwise comparisons when two values were calculated for each genome pair, the final ANIb was taken as the average of those two values. The results are presented as a plot, which was visualized in the R v.4.3.1 software environment [65] using the “pheatmap” package v.1.0.13.

## 3. Results

### 3.1. Short Description of Strain IMB-1

Gram-negative rods were visualized in the smear. Strain IMB-1 had the following biochemical properties: oxidase and catalase activity, hydrolyzed esculin, xylose, and arabinose, and possessed ß-glucosidase, α-galactosidase, ß-galactosidase, γ-glutamyltransferase, and phosphatase. Strain IMB-1 did not ferment carbohydrates and polyhydric alcohols (lactose, galactose, maltose, mannitol, sucrose, trehalose, cellobiose, inositol, and malonate), urease, and acetamide N-acetyl-ß-D-glucosaminidase; did not decarboxylate amino acids (arginine, ornithine, and lysine); and did not utilize citrate. The profile code 100022764, corresponding to the species *Chryseobacterium indologenes*, *Sphingomonas paucimobilis*, and *Vibrio hollisae*, was determined according to the NEFERMtest24 codebook.

### 3.2. Biochemical Properties of Strain IMB-1

Strain IMB-1 produced the following extracellular enzymes: it exhibited amylolytic activity, breaking down starch and creating a hydrolysis zone of 2–3 mm (Figure S1). Compared with the test strain *Corynebacterium kefirresidentii*, which had confirmed lipase activity, it weakly degraded lipids, as indicated by the formation of a barely visible opaque zone of calcium salts of fatty acids released from Tween around the streak (Figure S2). Strain IMB-1 lacked proteolytic activity and did not liquefy gelatin on MPG agar (Figure S3).

The IMB-1 strain formed a biofilm with a density of 0.075 ± 0.006 OD, which was 1.5 times higher than the OD value of the control (0.047).

### 3.3. Determination of the Antimicrobial Susceptibility Phenotype of Strain IMB-1

We used the SENSILA tests to identify the MIC and summarized the results obtained after 20 and 72 h of incubation because strain IMB-1 was slow-growing (Table S1).

Recommendations on antimicrobial susceptibility tests for groups of organisms or agents for which there are no EUCAST breakpoints were used for antibiotic testing. The MIC values for ceftazidime (8 mg/L), gentamicin (16 mg/L), amikacin (32 mg/L), cefotaxime (0.5 mg/L), cefepime (0.5 mg/L), ertapenem (2 mg/L), netilmicin (0.12 mg/L), and tobramycin (0.12 mg/L) were established (Table 1). According to a formal assessment by susceptibility category, the MIC values suggest that these agents should not be used for therapy.

The MIC values were also determined for ampicillin–sulbactam (1/0.5 mg/L), piperacillin (8 mg/L), piperacillin–tazobactam (2/4 mg/L), aztreonam (8 mg/L), meropenem (1 mg/L), colistin (0.25 mg/L), ciprofloxacin (0.12 mg/L), tigeciclin (0.06 mg/L), trimethoprim–sulfamethoxazole (0.06/1.19 mg/L), cefoperazone (16 mg/L), and cefoperazone–sulbactam (8/4 mg/L) (Table 1). A cautious interpretation suggests that these agents may be considered for therapy.

The simultaneous use of MIC NEFERM and MIC G-II kits to determine MIC allowed us to expand the range of antibiotics that can be used for the treatment of *Pseudoxanthomonas* infections. Despite the fact that there are no EUCAST breakpoints for assessing sensitivity to antibiotics for this microorganism, the use of experimentally determined MIC values can allow a cautious interpretation of the agents that may be considered for therapy and correctly calculate the dosage. Selected agents or their combinations may be suggested for the treatment of patients, taking into account their age, clinical diagnosis, and severity of the disease.

### 3.4. Identification of the IMB-1 Strain Using Mass Spectrometry

Clinical analysis using the MALDI-TOF mass spectrometric method classified strain IMB-1 as *Stenotrophomonas maltophilia* with a low confidence score. The IMB-1 strain and *S. maltophilia* strains 43 and 75 from the laboratory collection were retested to clarify these results. The two *S. maltophilia* strains were identified as *S. maltophilia* with values >2.35 and a convergence >94%, whereas for the IMB-1 strain, values ranged from 1.41 to 1.49 and a convergence from 49.76 to 52.59%, which is designated as “low identification confidence” in the MALDI results.

### 3.5. Ribosomal Taxonomy of Strain IMB-1

We conducted a phylogenetic analysis based on the 16S rRNA gene sequences of not only *Pseudoxanthomonas*-type species strains but also isolates not identified at the species level (Table S2). Ribosomal phylogeny revealed the polyphyleticity of *P. mexicana* and *P. kaohsiungensis* species (Figures S4 and S5).

Analysis of the description of data deposited in NCBI showed that bacteria belonging to the genus *Pseudoxanthomonas* are predominantly isolated from soil, bioreactors, and sludge, and less frequently from plants and their rhizosphere, water, and bioaerosols (Table S2). Single isolates were identified from biotopes associated with animals and humans. The 16S rRNA gene sequences of the isolates with medical origin were grouped into a cluster with the type strain *P. winnipegensis* NML 130738^T^ (Figure S4). Animal-associated isolates are predominantly clustered with strains of *P. winnipegensis*, *P. mexicana*, and *P. indica*, as well as in a separate cluster comprising *P. kaohsiungensis*, *P. koreensis*, *P. daejeonensis*, *P. suwonensis*, *P. broegbernensis*, and *P. jiangsuensis*.

The cluster formed by the type species *P. kaohsiungensis* includes sequences of isolates classified as *P. kaohsiungensis*, as well as those identified only to the genus level, the description of which indicates their isolation from natural biotopes, and *Pseudoxanthomonas* sp. strain EAG1 (KX839266.1) was isolated from the gut content of *Eisenia fetida* (Figure S5, Table S2).

### 3.6. Whole-Genome Sequencing of the IMB-1 Strain

Whole-genome sequencing of strain IMB-1 was performed using a combination of Oxford Nanopore long-read and Illumina short-read sequencing technologies. Nanopore sequencing generated long reads that enabled reconstruction of the chromosome structure, whereas Illumina reads were used for assembly polishing and correction of residual sequencing errors. Hybrid genome assembly, followed by iterative polishing, produced a high-quality complete genome sequence.

The final assembly consisted of a single circular chromosome of 3,671,183 bp with a GC content of 69.93%. The assembly exhibited high contiguity, with an N50 value equal to the chromosome length (3,671,183 bp), indicating the absence of fragmentation. Genome completeness reached 99.2% based on the gammaproteobacteria_odb10 dataset. The genome size and GC content were consistent with those reported for the type strain *Pseudoxanthomonas kaohsingensis* DSM 17583 (assembly ASM1021176 v1: 3,764,950 bp, GC 69.5%).

Genome annotation identified 3355 protein-coding sequences (CDSs), 50 tRNA genes, and 3 rRNA genes. The principal characteristics of the assembled genome are summarized in Table 2.

#### 3.6.1. Phylogenetic Analyses

The dDDH calculation based on the complete genome sequence showed that strain IMB-1 was closely grouped with the type strain *P. kaohsiungensis,* with the highest dDDH value of 70.1% (Table 3). With a species delineation threshold of 70% [66], strain IMB-1 belonged to *P. kaohsiungensis*.

**Figure 1:**
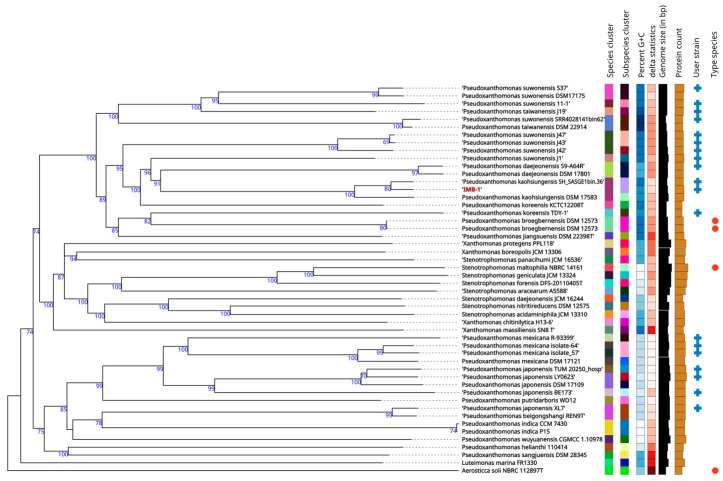
Tree inferred with FastME 2.1.6.1 [60] from GBDP distances calculated from genome sequences of bacteria of the genus *Pseudoxanthomonas*. The branch lengths are scaled in terms of the GBDP distance formula d5. The numbers above the branches represent the GBDP pseudo-bootstrap support values of >60% from 100 replications. The tree was rooted at the midpoint [61]. *P. kaohsiungensis* strain IMB-1 is marked in red font color.

The resulting phylogenetic tree revealed several species clusters for the genus *Pseudoxanthomonas*, one of which was assigned to the genome of the strain under study (Figure 1). The genomic cluster, together with the species *P. kaohsiungensis*, included species such as *P. suwonensis*, *P. daejeonensis*, *P. koreensis*, *P. broegbernensis*, and *P. taiwanensis*. Note the polyphyletic clustering of the whole genomes of *Pseudoxanthomonas* type species. Table S3 lists the resulting clusters of species and subspecies.

Additionally, we calculated the average nucleotide identity (ANI) and alignment fraction (AF) for the genomes of type and reference strains of the genus *Pseudoxanthomonas*, as well as for the studied strain IMB-1 (Table 4). Comparison of our strain with the *Pseudoxanthomonas* genomes consistently yielded values below 96% for all but *P. kaohsiungensis*, for which this value was 96.88%. These data confirm the results obtained using TYGS.

**Figure 2:**
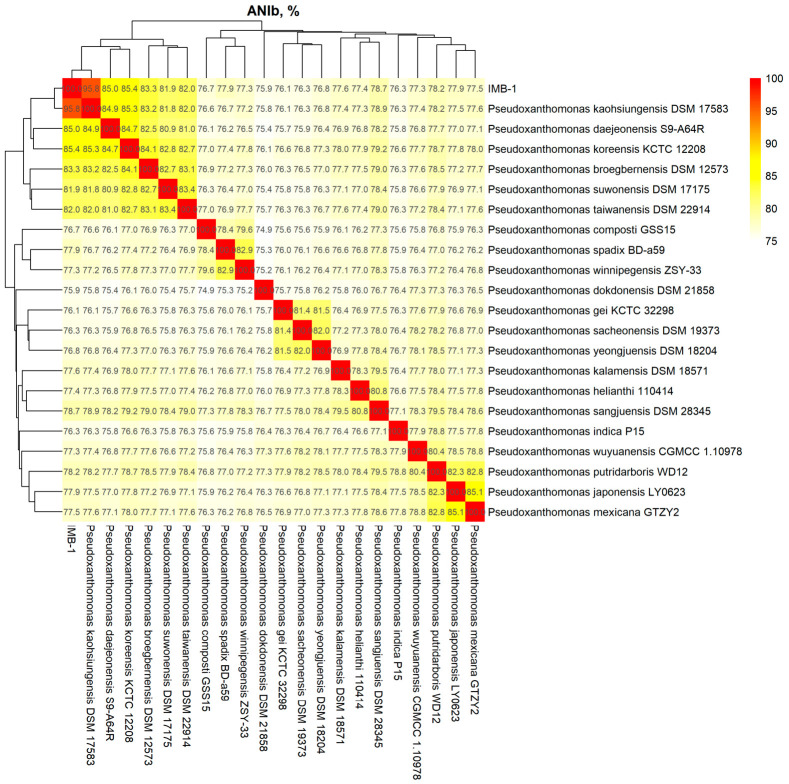
Heatmap presenting all pairwise, whole-genome sequence comparisons based on ANIb values obtained for the genome of strain IMB-1 and 21 reference genomes of *Pseudoxanthomonas* species type strains.

Finally, the WGS-based taxonomy was validated based on ANIb values and percentage coverage. At the time of writing, 23 validly published species have been listed on the LPSN website. However, the genomes of two species, *P. arseniciresistens* and *P. humi*, have not been deposited in the NCBI database. The final analysis included 21 reference genomes of valid *Pseudoxanthomonas* species. It has previously been shown that the recommended cutoff point of 70% DDH for species delineation corresponded to an ANI of 95 ± 0.5% [63]. Strain IMB-1 showed an ANI of 95.8% with the reference genome of the type strain, *P. kaohsiungensis* DSM 17583T, indicating species identification. Notably, the clustering of closely related species is consistent with both ribosome taxonomy and TYGS taxonomy and includes the species *P. kaohsiungensis*, *P. koreensis*, *P. daejeonensis*, *P. suwonensis*, *P. broegbernensis*, and *P. taiwanensis* (Figure 2). The aligned percentage was 74.72% for the closed-related genome of *P. kaohsiungensis* and <70% for the genomes of other *Pseudoxanthomonas* species (Figure 2).

#### 3.6.2. Features of Strain IMB-1’s Complete Genome

Functional annotation of the IMB-1 genome using eggNOG Mapper assigned the majority of predicted protein-coding genes to known COG functional categories (Figure S6). The largest category corresponded to genes of unknown function (S), accounting for approximately 20% of all annotated coding sequences. Among genes with assigned functions, the most abundant categories were amino acid transport and metabolism (E), cell wall/membrane/envelope biogenesis (M), transcription (K), replication, recombination and repair (L), translation, ribosomal structure and biogenesis (J), inorganic ion transport and metabolism (P), energy production and conversion (C), signal transduction mechanisms (T), and carbohydrate transport and metabolism (G). Genes involved in post-translational modification, lipid metabolism, coenzyme metabolism, intracellular trafficking, and cell motility were also identified, but represented a smaller proportion of the genome.

**Figure 3:**
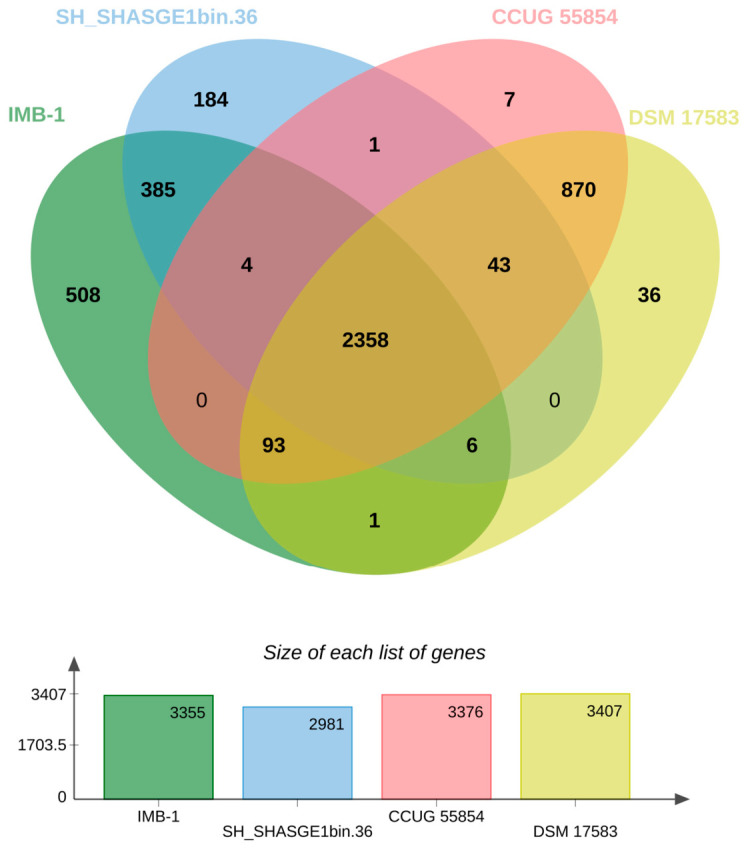
Venn diagram showing the distribution of shared and unique gene clusters among four *Pseudoxanthomonas kaohsiungensis* genomes: the clinical isolate IMB-1, the metagenome-assembled genome SH_SHASGE1bin.36, and two reference strains, CCUG 55854 and DSM 17583 (type strain). The diagram illustrates the size of the core genome (2358 gene clusters) shared by all strains, as well as strain-specific and accessory gene fractions. The bar chart below indicates the total number of gene clusters identified in each genome.

A comparative pan-genome analysis was performed for four *P. kaohsiungensis* genomes: the reference genome DSM 17583 assembly from type material; genome CCUG 55854 submitted by the Institution of Microbiology, Chinese Academy of Science; genome SH_SHASGE1bin.36 deposited as MIMAG metagenome-assembled genome (MAG) sample from *P. kaohsiungensis* (submitted by Xiamen University, China); and the studied genome IMB-1. The analysis revealed a substantial shared core genome comprising 2314 gene clusters, indicating a high level of genomic conservation within the strains (Figure 3). All four strains share hundreds of genes responsible for basic cellular processes: Translation and ribosomal proteins—most proteins of the 30S and 50S subunits (e.g., *rpsB/D/F/H/K/N/O/P/T/U*, *prmB*, *rplE/F/I/M/O/S/T/U/X/Y*, *rpmB/C/D/F/G/H/I*, *ykgO*). Metabolism—basic pathways for synthesizing amino acids (*proB*, *thrC*, *argB*), nucleotides *(purK*, *pyrH*), lipids (*cdsA*, *lpxL)*, and cofactors (*thi4*, *ubiI*). DNA replication and repair—genes *dnaN*, *recA*, *recQ*, *mutS*, and *uvrD*. Energy metabolism—genes encoding ATP synthase (*atpA/B/C/D/G/H*) and succinate dehydrogenase (*sdhA/B/C*) (Table S4).

Despite this conserved backbone, notable differences were observed in the accessory genome. The genome IMB-1 contained 508 unique gene clusters, representing the largest strain-specific gene set among the analyzed genomes. In comparison, the MAG SH_SHASGE1bin.36 harbored 184 unique genes, whereas genomes CCUG 55854 and DSM 17583 exhibited markedly smaller unique fractions (7 and 36 gene clusters, respectively). These differences suggest an increased genomic plasticity in genome IMB-1, potentially reflecting adaptation to the host-associated environment. The presence of heavy metal resistance genes and specific transport systems distinguished the IMB-1 genome from other genomes. Only the IMB-1 genome contains *cnrA* genes responsible for nickel and cobalt resistance, *cusR/S,* a copper-responsive two-component regulatory system component, and *copB* genes, ensuring resistance to toxic concentrations of heavy metals (copper/silver) and maintaining copper homeostasis in the cell. The gene *yhaV* (toxin) of the prokaryotic defense toxin–antitoxin system was found in the IMB-1 genome.

Pairwise and subgroup intersections further highlighted heterogeneity among the strains. Genome IMB-1 shared 385 gene clusters exclusively with SH_SHASGE1bin.36, whereas one and zero shared genes were observed between IMB-1 vs. the reference and IMB-1 vs. CCUG 55854, respectively. Notably, DSM 17583 and CCUG 55854 shared a relatively large number of gene clusters (870), consistent with their similar origin.

The intersection involving three genomes (excluding one) showed limited overlap, with only minor contributions (ranging from 4 to 93 gene clusters depending on the combination), indicating that most accessory genes are either strain-specific or shared within narrower phylogenetic or ecological groups. Only a single gene cluster was shared between SH_SHASGE1bin.36 and CCUG 55854, in the absence of other genomes, further emphasizing the divergence of the MAG from cultured reference strains.

Overall, the pan-genome structure is characterized by a large, conserved core and a diverse accessory genome, with the clinical strain IMB-1 exhibiting the highest proportion of unique genes. This pattern suggests ongoing genome diversification and may reflect niche-specific adaptations, including those associated with host colonization and potential pathogenicity.

Given the clinical significance of the studied strain, we analyzed resistance genes using CARD and ResFinder and virulence genes using the VFDB database as a reference.

Assessing the potential resistance of a clinically significant strain to antibiotics, disinfectants, and antiseptics is important to characterize its pathogenic properties. CARD analysis identified two genes with resistance potential in the IMB-1 strain genome. The specificity of the *fosI* gene was 70.0%, indicating its potential for phosphonic acid antibiotics resistance. The *vanY* gene in the *vanM* cluster showed a matching region identity of 32.37%, indicating a low degree of homology.

A ResFinder search for resistance genes yielded no positive results.

Additionally, pathways for classes such as Drug Resistance and Pathogenicity were identified in the genome annotated using KEGG. Although the completeness of some pathways is low, even though we analyzed the complete genome, the potential for implementing the full pathway may be quite high (Table 5). Of particular note is the presence of genes that ensure the functioning of the efflux pumps MexJK-OprM, MexAB-OprM, MexPQ-OpmE, AcrEF-TolC, MdtEF-TolC, and QacA, which mediate resistance to various classes of antibiotics.

**Figure 4:**
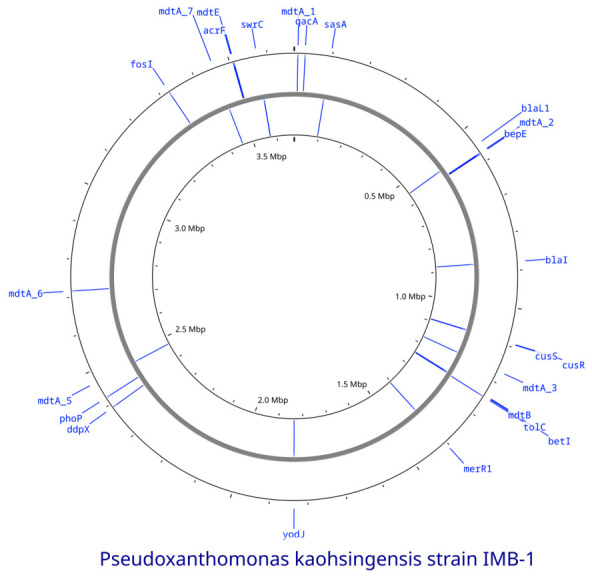
Circular genome map of *Pseudoxanthomonas kaohsiungensis* strain IMB-1. The ring represents the ordered genome, and the annotated features indicate the positions of predicted resistance genes, including antibiotic, disinfectant, and heavy metal resistance determinants.

A total set of ABR-associated genes was identified and mapped along the genome. These included genes associated with resistance to antibiotics, disinfectants, and heavy metals (Figure 4). The genome harbors genes encoding resistance to phosphonic acid antibiotics (*fosI*) and quaternary ammonium compounds (*qacL* and *qacA*), which is consistent with the phenotypic resistance profile observed in this strain. In addition, multiple genes related to multidrug efflux systems, including components of RND-type efflux pumps (*mdtA*, *mdtB*, *mdtE*, *acrF*, and *swrC*), were detected, suggesting the presence of multidrug resistance mechanisms.

Furthermore, genes associated with resistance to β-lactam antibiotics (*blaI* family regulators and related elements) were identified, alongside regulatory systems such as *phoP*, which contribute to stress response and antimicrobial tolerance. The genome also contains several genes, including *cusS*, *cusR*, and *cueR*, linked to resistance to toxic compounds and metals, indicating potential adaptation to harsh environmental conditions.

The spatial distribution of these genes across the genome appears dispersed rather than clustered into a single resistance island, suggesting multiple independent acquisition or ancestral retention events. The presence of both antibiotic resistance determinants and stress-response systems supports the hypothesis that strain IMB-1 possesses a broad adaptive potential, which may facilitate survival in both environmental and host-associated niches.

Table S5 provides a complete list of antibiotic resistance genes identified along with their genomic coordinates and predicted functions.

The genome of strain IMB-1 contained genes of the following classes, which were identified using the VFDB database: Adherence, Motility, and Others (Isocitrate lyase) (Table S6). The presence of gene clusters indicates the ability of the strain to form biofilms, adhere to the epithelial surface, and exhibit resistance to stress factors.

Notably, the complete genome of the *P. kaohsiungensis* strain IMB-1 lacked plasmid and prophage sequences.

### 3.7. Comparative Analysis of Phenotypic Resistance Profiles and Genetic Markers in the Genome of P. kaohsiungensis Strain IMB-1

**Figure 5:**
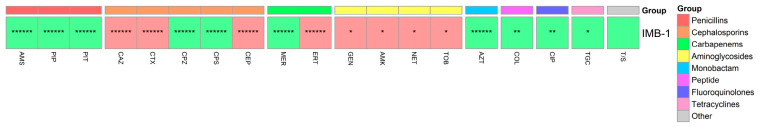
Comparison of phenotypic susceptibility profiles and genetic determinants of resistance in *Pseudoxanthomonas kaohsiungensis* strain IMB-1. AMS—ampicillin/sulbactam; PIP—piperacillin; PIT—piperacillin/tazobactam; CAZ—ceftazidime; CTX—cefotaxime; CPZ—cefoperazone; CPS—cefoperazone/sulbactam; CEP—cefepime; MER—meropenem; ERT—ertapenem; GEN—gentamicin; AMK—amikacin; NET—netilmicin; TOB—tobramycin; AZT—aztreonam; COL—colistin; CIP—ciprofloxacin; TGC—tigecycline; T/S—trimethoprim/sulfamethoxazole. Susceptibility to antibiotics is marked in green, and resistance to antibiotics is marked in red. The presence of genetic determinants is indicated by black asterisks; the number of asterisks corresponds to the number of detected genes (Table S5).

A comparative study of phenotypic susceptibility patterns and genetic resistance determinants in *P. kaohsiungensis* strain IMB-1 revealed both overall concordance and inconsistencies between phenotypic resistance profiles and genetic markers (Figure 5). Phenotypic resistance to gentamicin, amikacin, netilmicin, and tobramycin corresponded to the presence of aminoglycoside resistance genes. The susceptibility of the IMB-1 strain to trimethoprim–sulfamethoxazole was also correlated with the absence of detectable genetic determinants of resistance to these antibiotics. However, the discrepancies between genotype and phenotype were identified. Despite the presence of genetic determinants associated with antibiotic resistance in the IMB-1 genome, phenotypic variability was observed. For example, genes for resistance to beta-lactam antibiotics were detected in the genome, but the strain was susceptible to ampicillin–sulbactam, piperacillin, piperacillin–tazobactam, cefoperazone, cefoperazone–sulbactam, and meropenem. A similar situation occurred with monobactams, fluoroquinolones, and tetracyclines: the strain remained susceptible to these antibiotics despite the presence of relevant resistance genes. Resistance mechanisms may be used if genetic resistance determinants are present. This allows us to evaluate the risk of horizontal gene transfer and the spread of resistance to other microorganisms.

A comprehensive analysis of the genomes of opportunistic bacteria, including the search for antibiotic resistance determinants and an assessment of their localization, is essential for understanding the molecular mechanisms of antibiotic resistance and the epidemiological potential of rare pathogens. Studying bacterial genomes using molecular genetic methods and bioinformatics analysis enables the identification of patterns in the microevolution of opportunistic bacteria and predicts the risks of antibiotic resistance.

## 4. Discussion

Among the species of the genus *Pseudoxanthomonas*, it is worth noting that only strains of the species *P. winnipegensis* were originally derived from human clinical materials [17]. To date, only individual strains of the species *P. mexicana* and *P. kaohsiungensis*, which were isolated from patients’ urine and blood, have been reported by the authors to be involved in the infectious process [1,14]. Representatives of this genus have been associated with various diseases as microbiota-associated markers of human diseases, as detected by next-generation sequencing methods [19,20,21]. This indicates the clinical significance of these bacteria and the need for a thorough study of their properties for the accurate and rapid diagnosis of *Pseudoxanthomonas* infections.

In 2005, the type species *P. kaohsiungensis* strain J36^T^ was isolated from an oil-polluted site in southern Taiwan near Kaohsiung City [15]. The authors characterized this strain as a biosurfactant-producing bacterium. Thirteen years later, a slow-growing isolate was isolated from the patient’s blood and identified as *P. kaohsiungensis* [1]. This oil refinery worker in Kaohsiung, Taiwan, had been exhibiting symptoms indicative of a chronic infection caused by a low-virulence pathogen for over three months. The authors concluded that *P. kaohsiungensis* is not simply a bacterium producing biosurfactants in the environment but a potential human pathogen in immunocompromised individuals [1]. The One Health concept is currently a key global approach to addressing complex human, animal, and environmental health issues [67]. Interdisciplinary collaboration is becoming evident with the growing threat of antimicrobial resistance caused by the overuse of antibiotics in humans, veterinary, and agricultural medicine [68]. Climate change, loss of biodiversity, degradation of ecosystems, and anthropogenic pressures intensify epidemiological risks, contributing to the emergence of new pathogens and the expansion of the ranges of existing ones. Among the phenomena contributing to the emergence of new pathogens, refining the list of Botvinkin A.D. and co-authors, compiled 25 years ago, we can highlight the following, which are currently applicable in bacteriology: (1) the discovery of new species in known taxa of microorganisms; (2) the identification of etiological agents previously considered non-infectious; (3) the diversity of forms associated with circulation in different hosts; (4) natural variability as the main survival strategy of microorganisms; (5) variability induced by industrialization and humans [69].

We present the identification of strain IMB-1, previously isolated from a child’s cerebrospinal fluid, based on ribosomal taxonomy and phylogenomic analysis according to whole-genome sequencing. The biological properties, resistance phenotype, and key virulence and resistance factors based on genomic data have been described. *P. kaohsiungensis* strain IMB-1 displayed amylolytic and weak lipolytic activities, and it exhibited a low phenotypic resistance profile regarding antibiotic susceptibility. Resistance potential can be noted only for aminoglycosides according to the MIC values. We compared the phenotypic resistance profiles of species included in the same genomic cluster with *P. kaohsiungensis* and species *P. winnipegensis* and *P. mexicana* (Table 6).

Overall, the resistance phenotypes presented for the studied strains indicate their susceptibility to most of the antibiotics tested. However, bacterial genomes are highly flexible and possess diverse regulatory mechanisms that bacteria use to survive stressful conditions. Identifying clinically important genes in the genomes of opportunistic pathogenic bacteria or environmental isolates and studying the regulatory mechanisms of their expression under stressful conditions can help develop approaches to preventing the emergence of additional resistance genes in clinical practice.

Two modern technologies, mass spectrometry and high-throughput sequencing, allow for the implementation of breakthrough diagnostic approaches in clinical bacteriology, contributing to increased accuracy of pathogen identification and the speed of diagnostic procedures.

Matrix-assisted laser desorption/ionization time-of-flight mass spectrometry (MALDI-TOF MS) is used in clinical diagnostics to identify microorganisms of various genera and species, as well as to identify strains within a species. A database is used to compare the mass spectra of unknown microorganisms with those of reliably identified microorganisms. During the comparison, a convergence coefficient is calculated based on the correlation between the obtained peaks and their intensities. MALDI-TOF MS is more effective in identifying bacteria and fungi than most biochemical tests [73,74,75,76]. However, this approach’s clinical use for identifying rare pathogens has limitations, primarily related to the lack of reference spectra in the database. Recent studies have shown that MALDI-MS is highly sensitive, and the identification and characterization of biomarker peaks allows for the discrimination of bacteria at the species level. For example, Takei et al. demonstrated that peaks of L29, L33, and CspA are useful for distinguishing *Pseudoxanthomonas* at the species level [72].

Whole-genome sequencing (WGS) provides information on test strain species, serotype, virulence, and antimicrobial resistance genotypes [4,77]. We have proposed a high resistance potential for strain IMB-1 due to the presence of genes encoding porin proteins, efflux pumps, resistance to heavy metals, and specific transport systems. Identification of genes encoding the QacA efflux pump suggested a potential resistance to disinfectants and antiseptics. Genomic data analysis indicated virulence potential, which was confirmed by the presence of the type I secretion system (TISS; KEGG module M00575), type IV pili, genes responsible for flagellar biosynthesis, and isocitrate lyase (ICL). TISS is responsible for the export of toxins, such as adenylate cyclase (CyaA), which facilitate colonization, suppress the immune response, and promote disease progression [78]. Subclass of type IV pili mediate biofilm formation, predation, and surface sensing in many bacteria, and their crucial function is host cell adherence as an initial step in colonization [79]. ICL allows pathogens to utilize non-glucose carbon sources via catalyzes the cleavage of isocitrate into succinate and glyoxylate to survive and replicate [80]. The application of high-resolution WGS data in public health for pathogen identification and monitoring can improve the accuracy of infection source determination, reduce the scale and burden of outbreaks, and identify and quantify antimicrobial resistance in pathogen strains [81]. Reducing the cost of WGS, increasing the technology’s speed and accuracy, and advances in bioinformatics are critical to its large-scale and effective use for pathogen surveillance.

The need for further experimental evidence on the clinical significance of the newly isolated pathogen is a limitation of this study. Another limitation is that only one clinical strain was analyzed in this study. More data on clinical isolates of *Pseudoxanthomonas* species should be collected. We note the insufficient knowledge, difficulty in identification, and potential underestimation of *P. kaohsiungensis*’ contribution to disease development and dynamics.

## 5. Conclusions

Our study demonstrates that the *P. kaohsiungensis* strain IMB-1 is a potential opportunistic pathogen with significant pathogenic potential. The misidentification of the IMB-1 strain using biochemical tests and MALDI-TOF MS highlights the importance of genomic tools for accurate pathogen identification and characterization. These results highlight the urgent need for enhanced genomic monitoring, updated microbial identification databases, and ongoing drug-resistant pathogen surveillance. These efforts are essential to mitigate the growing threat of antimicrobial resistance and improve infection control strategies.

## Figures and Tables

**Table 1:** Evaluation of the MIC values of antimicrobial agents against the *P. kaohsiungensis* strain IMB-1 that does not have EUCAST breakpoints.

SENSILA Test NEFERM		SENSILA Test G II	
Name of Antibiotics	MIC, mg/L	Name of Antibiotics	MIC, mg/L
Ampicillin/sulbactam	1/0.5	Cefotaxime	0.5
Piperacillin	8	Cefoperazone	16
Piperacillin/tazobactam	2/4	Cefoperazone/sulbactam	8/4
Ceftazidime	8	Cefepime	0.5
Aztreonam	8	Ertapenem	2
Meropenem	1	Netilmicin	0.12
Gentamicin	16	Tobramycin	0.12
Amikacin	32		
Colistin	0.25		
Ciprofloxacin	0.12		
Tigecycline	0.06		
Trimethoprim/sulfamethoxazole	0.06/1.19		

**Table 2:** Assembly and annotation statistics of the complete genome of strain IMB-1.

Indicators	IMB-1
**Genome assembly**	
Sequencing strategy	Hybrid
Chromosome topology	Circular
Number of contigs	1
Genome size, bp	3,671,183
N50, bp	3,671,183
GC, %	69.93
Completeness, %	99.2
**Genome annotation**	
Number of CDSs	3355
Number of rRNA genes	3
Number of tRNA genes	50

bp—base pairs; CDSs—coding DNA sequences; rRNA—ribosomal ribonucleic acid; tRNA—transfer ribonucleic acid.

**Table 3:** Digital DNA–DNA hybridization of genomes to compare strain IMB-1 to closely related type and reference strains belonging to the genus *Pseudoxanthomonas* according to TYGS analysis.

Subject Strain	DDH (d4, in %)	G + C Content Difference (in %)
*P. kaohsiungensis* DSM 17583^T^	70.1	0.21
*P. koreensis* KCTC 12208^T^	29.4	0.25
*P. daejeonensis* DSM 17801^T^	28.9	1.04
*P. jiangsuensis* DSM 22398^T^	27.4	0.50
*P. broegbernensis* DSM 12573^T^	26.7	0.74
*P. suwonensis* DSM 17175^T^	24.9	0.46
*P. taiwanensis* DSM 22914^T^	24.6	2.14
*P. sangjuensis* DSM 28345^T^	22.4	1.25
*P. japonensis* DSM 17109^T^	21.3	2.62
*P. indica* CCM 7430^T^	21.0	4.49

TYGS—Type (Strain) Genome Server; DDH—DNA–DNA hybridization.

**Table 4:** ANI and AF values of the IMB-1 genome compared with those of the most closely related and reference *Pseudoxanthomonas* strains (%).

Species Name	ANI	AF
*P. kaohsiungensis* DSM 17583^T^	96.88	84.61
*P. koreensis* KCTC 12208^T^	87.76	73.15
*P. daejeonensis* DSM 17801^T^	87.66	79.22
*P. jiangsuensis* DSM 22398^T^	87.28	72.76
*P. broegbernensis* DSM 12573^T^	86.16	68.69
*P. suwonensis* DSM 17175^T^	85.69	71.38
*P. taiwanensis* DSM 22914^T^	85.39	70.24
*P. sangjuensis* DSM 28345^T^	83.09	59.85
*P. japonensis* DSM 17109^T^	81.94	58.19
*P. indica* CCM 7430^T^	80.88	50.99

ANI—average nucleotide identity; AF—alignment fraction.

**Table 5:** Pathways within the Drug Resistance and Pathogenicity classes detected in the complete genome of the *Pseudoxanthomonas kaohsiungensis* strain IMB-1.

**Module Accession**	**Completeness**	**Pathway Name**	**Matching KO**	**Missing KO**
**Signature modules; Gene set; Drug resistance**				
M00745	100.0	Imipenem resistance, repression of porin OprD	K07644, K07665	-
M00642	75.0	Multidrug resistance, efflux pump MexJK-OprM	K18301, K18303	K18302
M00744	66.67	Cationic antimicrobial peptide (CAMP) resistance, protease PgtE	K07637, K07660	K08477
M00714	50.0	Multidrug resistance, efflux pump QacA	K08167	K18938
M00627	33.33	Beta-lactam resistance, Bla system	K02171	K02172, K18766
M00718	33.33	Multidrug resistance, efflux pump MexAB-OprM	K03585, K18138	K18131, K18139
M00769	33.33	Multidrug resistance, efflux pump MexPQ-OpmE	K19591	K18304, K19593, K19594, K19595
M00651	25.0	Vancomycin resistance, D-Ala-D-Lac type	K07260	K18344, K18345, K18346
M00696	16.67	Multidrug resistance, efflux pump AcrEF-TolC	K12340	K18140, K18141, K18142
M00697	16.67	Multidrug resistance, efflux pump MdtEF-TolC	K12340	K07690, K18898, K18899
**Signature modules; Gene set; Pathogenicity**				
M00575	20.00	Pertussis pathogenicity signature, T1SS	K12340	K07389, K11003, K11004, K22944

KO—Database Identifier KEGG Orthology.

**Table 6:** Phenotypic resistance profile of species included in the genomic cluster with *P. kaohsiungensis,* as well as the profile of species *P. winnipegensis* and *P. mexicana*, derived from human clinical sources.

Name	Isolation Source	Location	Method/Antimicrobial Susceptibility	Reference
*P. kaohsiungensis* strain J36^T^	Oil-polluted site	Kaohsiung City in southern Taiwan	Disk diffusion: Resistant to amikacin, gentamicin, kanamycin, and streptomycin. Susceptible to ampicillin, cefotaxime, chloramphenicol, nalidixic acid, rifampin, streptomycin, and tetracycline	[15]
*P. kaohsiungensis*	Blood culture	Kaohsiung City in southern Taiwan	The authors did not provide MIC values. They reported that the patient was treated with ceftazidime and ciprofloxacin, and his condition improved	[1]
*P. kaohsiungensis* strain IMB-1	Cerebrospinal fluid	Irkutsk, Russia	MIC values: High values for ceftazidime, gentamicin, amikacin, cefotaxime, cefepime, ertapenem, netilmicin, and tobramycin. Low values for ampicillin–sulbactam, piperacillin, piperacillin–tazobactam, aztreonam, meropenem, colistin, ciprofloxacin, tigeciclin, trimethoprim–sulfamethoxazole, cefoperazone, and cefoperazone–sulbactam	Recent study
*P. koreensis* strain T7-09^T^	Soil from a ginseng field	South Korea	No data	[70]
*P. daejeonensis* strain TR6-08^T^	Soil from a ginseng field	South Korea	No data	[70]
*P. broegbernensis* strain B1616/1^T^	Biofilters	Germany	Disk diffusion: Resistant to erythromycin, streptomycin, nalidixic acid, kanamycin, ampicillin, penicillin G, gentamicin, fucidin, tetracycline, and novobiocin. Susceptible to neomycin	[12]
*P. suwonensis* strain 4M1^T^	Cotton waste composts	Korea	No data	[71]
*P. taiwanensis* strain CB-226^T^	Chi-ban Hot Springs	Eastern Taiwan	No data	[13]
*P. winnipegensis* strain NML 130738^T^; a total of 12 isolates	10 cystic fibrosis/other patient types and a variety of clinical sources	Canada	MIC values: All strains had high MICs towards nitrofurantoin. Intermediate MIC values: Resistant for some strains for meropenem and imipenem. Low values: Susceptible to amikacin, aztreonam, cefepime, ceftriaxone, ceftazidime, ciprofloxacin, gatifloxacin, gentamicin, piperacillin, piperacillin/taxobactam, ticarcillin/clavulanic acid, and tobramycin	[17]
*P. winnipegensis* strain JUPW001	Blood culture	Tokyo, Japan	The authors did not provide MIC values. They reported that the patient was treated with piperacillin/tazobactam, and her condition improved	[72]
*P. mexicana* strain AMX 26B^T^	Anaerobic digester	Mexico	MIC values: High values for aminoglycosides and pipemidic acid. Intermediate values for fusidic acid and erythromycin. Low values for doxycycline, colistin, fluoroquinolones, carbapenems, cephems, and penams	[14]
*P. mexicana* strain UR374_02	Human urine	Mexico	MIC values: High values for amikacin, kanamycin, netilmicin, and tobramycin. Intermediate values for pipemidic acid and penicillin G. Low values for gentamicin, fusidic acid, erythromycin, doxycycline, colistin, fluoroquinolones, carbapenems, cephems, and penams	[14]

## Data Availability

The original contributions presented in this study are included in the article material. Further inquiries can be directed to the corresponding author. The draft and complete genomes of the *P. kaohsiungensis* strain IMB-1 have been uploaded to the NCBI database (Bioproject PRJNA1443677).

## Supplementary Materials

The following supporting information can be downloaded at https://www.mdpi.com/article/10.3390/biology15131010/s1. Figure S1: Visualization of amylolytic activity on a starch-containing medium after treatment with Lugol’s iodine solution: (A) positive reaction of strain IMB-1; (B) negative reaction of *S. aureus* ATCC 25923. The starch-containing medium turned blue. The starch hydrolysis zone was measured in millimeters from the edge of the streak to the edge of the light zone. Figure S2: Visualization of lipolytic activity on brain heart agar: (A) strain IMB-1; (B) *Corynebacterium kefirresidentii*. Exogenous lipase activity was assessed by the formation of a “halo” of insoluble calcium salts of free fatty acids around the colonies. Figure S3: Visualization of proteolytic activity of strain IMB-1 on meat-peptone gelatin. No liquefaction of gelatin during sowing by injection indicates a lack of activity. Figure S4: Ribosomal taxonomy of bacteria of the genus *Pseudoxanthomonas*. Figure S5: Subtree of phylogenetic analysis of 16S rRNA genes of bacteria genus *Pseudoxanthomonas.* 16S rRNA gene of *P. kaohsiungensis* strain IMB-1 is marked in bold; bootstrap values higher than 75% are shown; scale bar represents one substitution per 100 bp. Figure S6: Predicted protein-coding genes of *Pseudoxanthomonas kaohsiungensis* strain IMB-1 among COG functional categories according to eggNOG Mapper annotation. Table S1. Susceptibility to antibiotics and MIC of antibiotics for strain IMB-1 obtained by two tests. Table S2: Brief description of the 16S rRNA gene sequence data of strains, isolates, and clones deposited in NCBI, including those not identified to species, used for ribosomal taxonomy. Table S3. The resulting species and subspecies clusters based on dDDH according to TYGS analysis. Table S4. Shared and unique gene clusters among four *Pseudoxanthomonas kaohsiungensis* genomes: reference genome DSM 17583 assembly from type material, genome CCUG 55854, MAG SH_SHASGE1bin.36, and studied genome IMB-1. Table S5. A complete list of antibiotic resistance genes identified in the complete genome of the *Pseudoxanthomonas kaohsiungensis* strain IMB-1, along with their genomic coordinates and predicted functions. Table S6. Virulence factors found in the complete genome of *Pseudoxanthomonas kaohsiungensis* strain IMB-1.

## Abbreviations

The following abbreviations are used in this manuscript:

AF	Alignment Factors
ANI	Average Nucleotide Identity
BHA	Brain Heart Agar
BLAST	Basic Local Alignment Search Tool
bp	Base Pairs
CDSs	Coding DNA Sequences
dDDH	Digital DNA–DNA Hybridization
DNA	Deoxyribonucleic Acid
GBDP	Genome BLAST Distance Phylogenies
KEGG	Kyoto Encyclopedia of Genes and Genomes
KO	Database Identifier KEGG Orthology
LPSN	List of Prokaryotic Names with Standing in Nomenclature
MALDI-TOF	Matrix-Assisted Laser Desorption/Ionization Time-of-Flight
MIC	Minimum Inhibitory Concentration
MPG	Meat-Peptone Gelatin
NCBI	National Center for Biotechnology Information
NFGNB	Non-Fermenting Gram-Negative Bacteria
NJ	Neighbor-Joining
PCR	Polymerase Chain Reaction
rRNA	Ribosomal RNA
SC FHHRP	Scientific Center for Family Health and Human Reproduction Problems
T1SS	Type I Secretion Systems
tRNA	Transfer RNA
TYGS	Type (Strain) Genome Server
VFDB	Virulence Factors Database
WGS	Whole-Genome Sequencing
