# Supplementary material for: Pathogenic Potential of Pseudoxanthomonas kaohsiungensis Strain IMB-1 Based on Whole-Genome Sequencing
Source: Biology (Basel). 2026 Jun 25;15(13):1010. doi: 10.3390/biology15131010 (PMC13359763; doi:10.3390/biology15131010)
Supplement: Supplementary file 1 [file biology-15-01010-s001.zip › Figures S1-S3.pdf]

A B

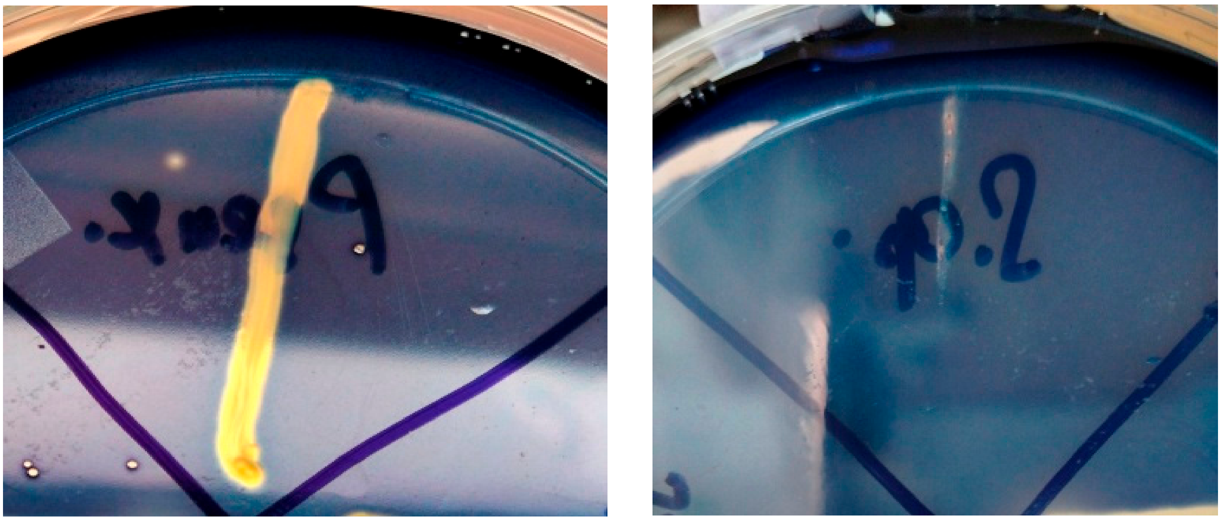

Figure S1. Visualization of amylolytic activity on a starch-containing medium after treatment with Lugol's iodine solution: (A) positive reaction of strain IMB-1; (B) negative reaction of *S. aureus* ATCC 25923. The starch-containing medium turned blue. The starch hydrolysis zone was measured in millimeters from the edge of the streak to the edge of the light zone.

A B

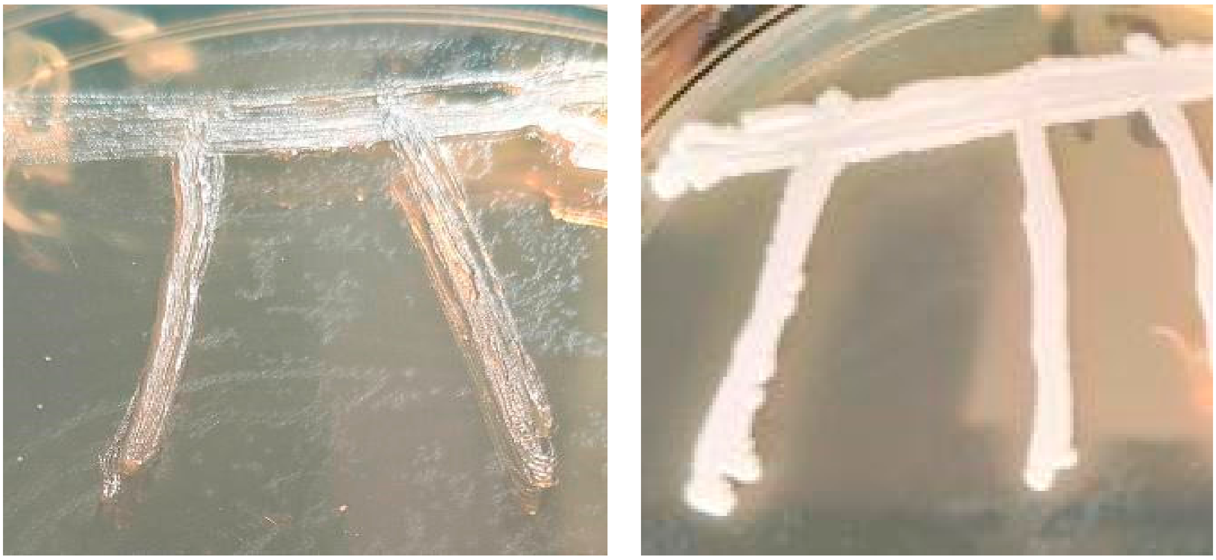

Figure S2. Visualization of lipolytic activity on brain heart agar: (A) – strain IMB-1; (B) – *Corynebacterium kefirresidentii*. Exogenous lipase activity was assessed by the formation of a "halo" of insoluble calcium salts of free fatty acids around the colonies.

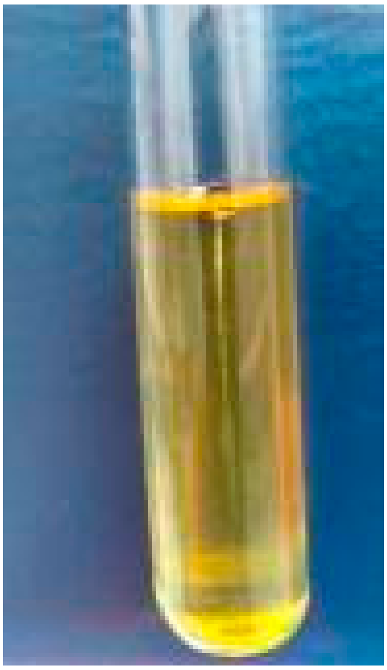

Figure S3. Visualization of proteolytic activity of strain IMB-1 on meat-peptone gelatin. No liquefaction of gelatin during sowing by injection indicates a lack of activity.
