# Supplementary figures and images for: Pathogenic Potential of Pseudoxanthomonas kaohsiungensis Strain IMB-1 Based on Whole-Genome Sequencing
Source: Biology (Basel). 2026 Jun 25;15(13):1010. doi: 10.3390/biology15131010 (PMC13359763; doi:10.3390/biology15131010)

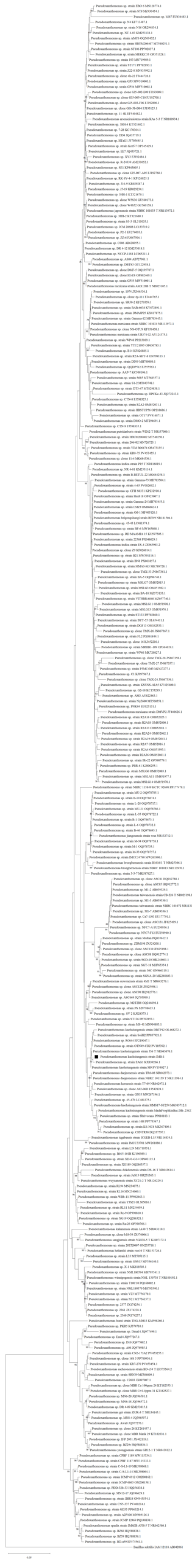

Supplement: Supplementary file 1 [file biology-15-01010-s001.zip › Figure S4.png]

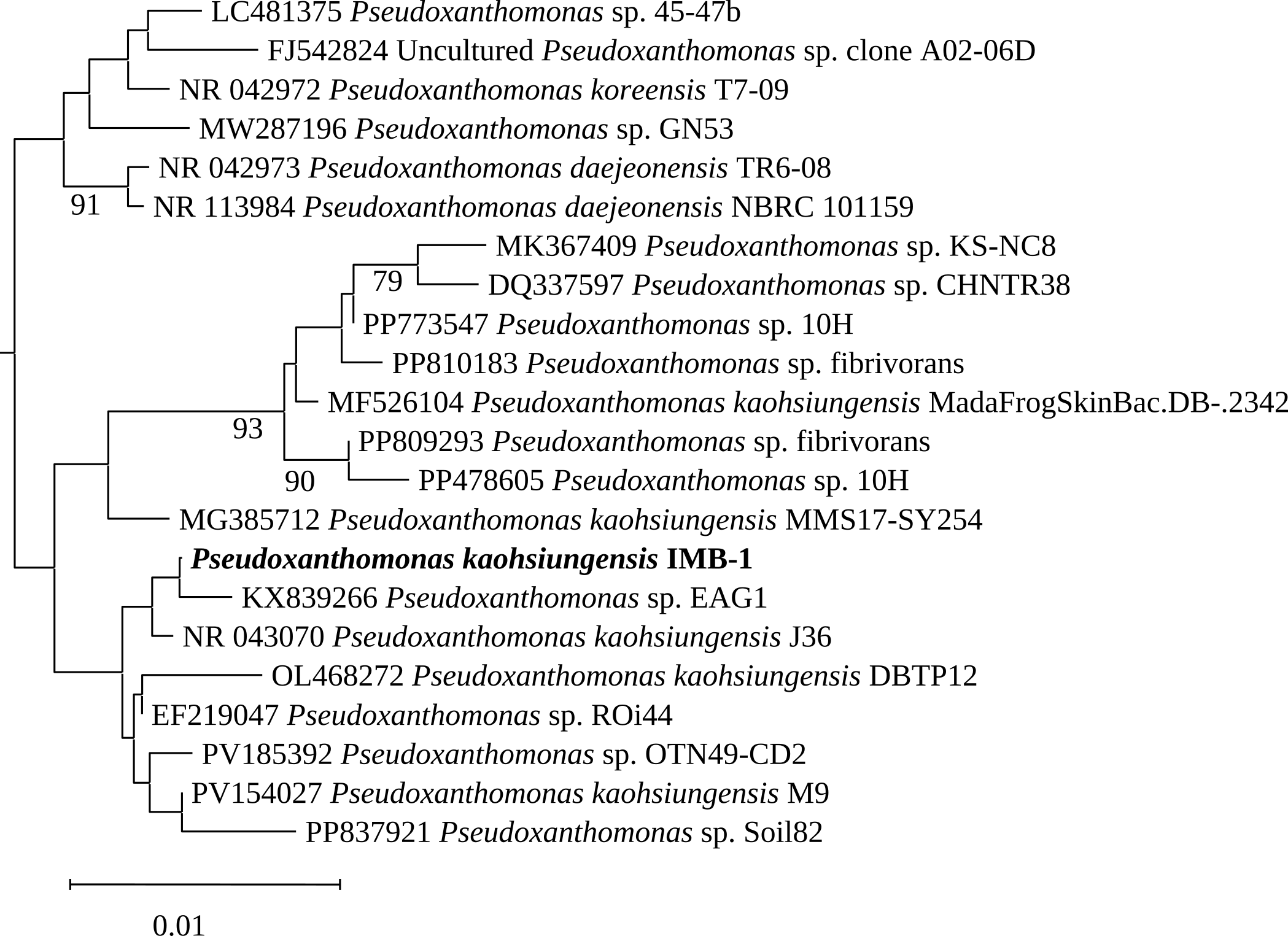

Supplement: Supplementary file 1 [file biology-15-01010-s001.zip › Figure S5.tiff]

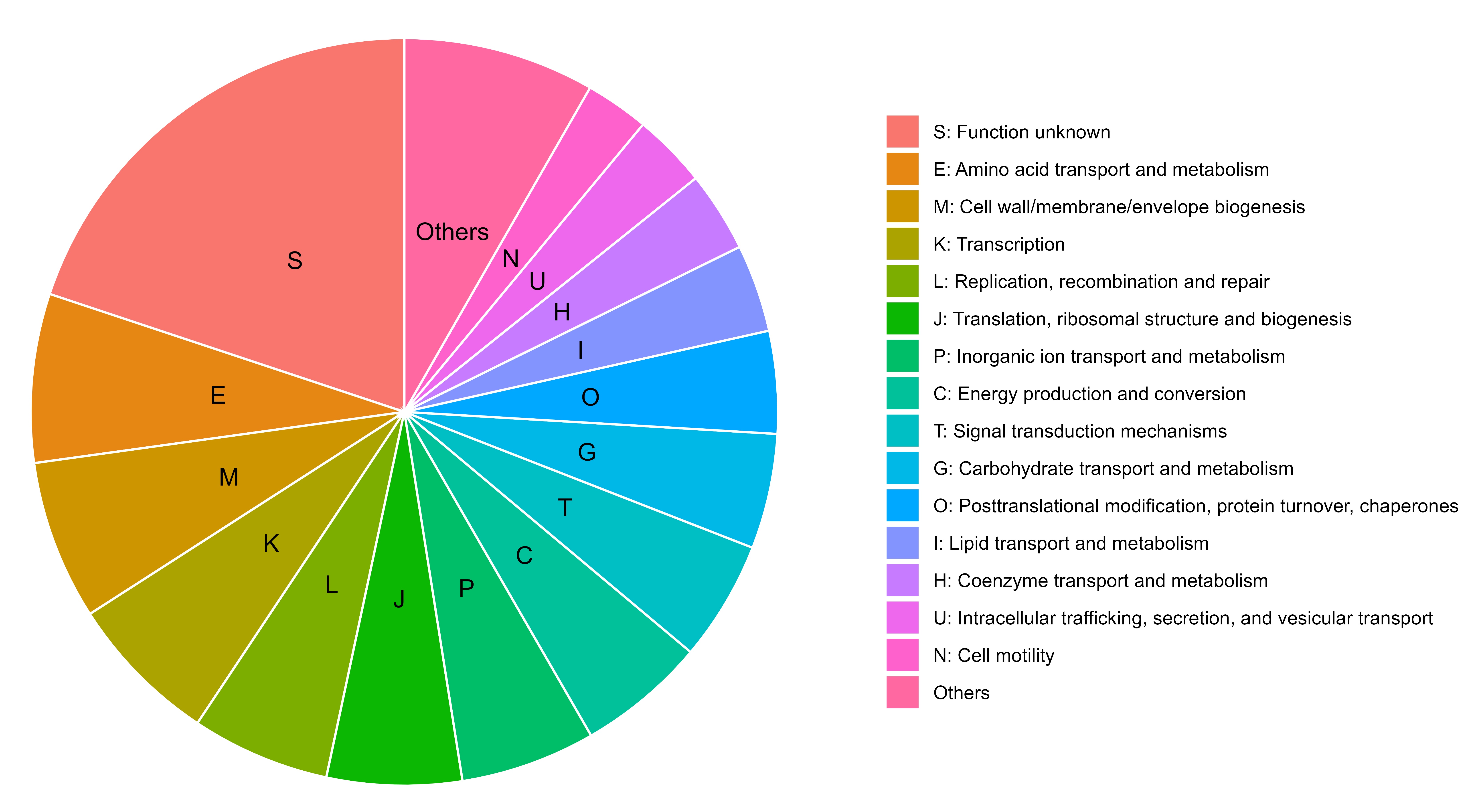

Supplement: Supplementary file 1 [file biology-15-01010-s001.zip › Figure S6.jpg]
